# A comparative evaluation of mobile medical APPS (MMAS) for reading and interpreting malaria rapid diagnostic tests

**DOI:** 10.1186/s12936-020-03573-2

**Published:** 2021-01-13

**Authors:** Theodoor Visser, Sumedh Ramachandra, Emilie Pothin, Jan Jacobs, Jane Cunningham, Arnaud Le Menach, Michelle L. Gatton, Samaly dos Santos Souza, Sydney Nelson, Luke Rooney, Michael Aidoo

**Affiliations:** 1 Clinton Health Access Initiative, Boston, USA; 2 Swiss Tropical and Public Health Institute, Basel, Switzerland; 3 Institute of Tropical Medicine, Antwerp, Belgium; 4 Department of Microbiology and Immunology, KU Leuven, Leuven, Belgium; 5 World Health Organization, Geneva, Switzerland; 6 Queensland University of Technology, Brisbane, Australia; 7 Centers for Disease Control and Prevention, Atlanta, USA; 8 Georgia Institute of Technology, Atlanta, USA

**Keywords:** Malaria, Rapid Diagnostic Test, RDT, Reader, Diagnosis, Mobile medical application, App, Diagnostics, mHealth, Sensitivity, Specificity, Accuracy

## Abstract

**Background:**

The World Health Organization recommends confirmatory diagnosis by microscopy or malaria rapid diagnostic test (RDT) in patients with suspected malaria. In recent years, mobile medical applications (MMAs), which can interpret RDT test results have entered the market. To evaluate the performance of commercially available MMAs, an evaluation was conducted by comparing RDT results read by MMAs to RDT results read by the human eye.

**Methods:**

Five different MMAs were evaluated on six different RDT products using cultured *Plasmodium falciparum* blood samples at five dilutions ranging from 20 to 1000 parasites (p)/microlitre (µl) and malaria negative blood samples. The RDTs were performed in a controlled, laboratory setting by a trained operator who visually read the RDT results. A second trained operator then used the MMAs to read the RDT results. Sensitivity (Sn) and specificity (Sp) for the RDTs were calculated in a Bayesian framework using mixed models.

**Results:**

The RDT Sn of the *P. falciparum* (Pf) test line, when read by the trained human eye was significantly higher compared to when read by MMAs (74% vs. average 47%) at samples of 20 p/µl. In higher density samples, the Sn was comparable to the human eye (97%) for three MMAs. The RDT Sn of test lines that detect all *Plasmodium* species (Pan line), when read by the trained human eye was significantly higher compared to when read by MMAs (79% vs. average 56%) across all densities. The RDT Sp, when read by the human eye or MMAs was 99% for both the Pf and Pan test lines across all densities.

**Conclusions:**

The study results show that in a laboratory setting, most MMAs produced similar results interpreting the Pf test line of RDTs at parasite densities typically found in patients that experience malaria symptoms (> 100 p/µl) compared to the human eye. At low parasite densities for the Pf line and across all parasite densities for the Pan line, MMAs were less accurate than the human eye. Future efforts should focus on improving the band/line detection at lower band intensities and evaluating additional MMA functionalities like the ability to identify and classify RDT errors or anomalies.

## Background

Malaria rapid diagnostics tests (RDT) are lateral flow assays (LFAs) that detect malaria specific antigens produced by the parasites. Malaria RDTs can detect either a single species (either *Plasmodium falciparum* or *Plasmodium vivax*), or multiple (Pan) species (*P. falciparum, P. vivax, Plasmodium malariae* or *Plasmodium ovale*). Malaria RDTs detecting both a *P. falciparum* and a pan-antigen are commonly called combination (‘combo’) RDTs. The World Health Organization (WHO) recommends use of quality-assured RDTs (or microscopy) in *all* patients suspected of malaria [1, 2]. Between 2010 and 2018, the proportion of suspected malaria cases receiving a parasitological test among patients presenting for care in the public sector in the WHO African region increased from 36 to 85% [3]. This increase has mainly been driven by the use of RDTs, which accounted for 75% of diagnostic testing among suspected cases in 2017 [4].

Malaria RDTs are relatively easy to use and provide a result within a short period of time (i.e., 15 or 20 mins). Malaria RDTs are used at all levels of the healthcare system including community health worker networks [5]. They are typically performed by collecting a finger prick blood sample from the patient and transferring it to a sample well on the test card along with certain reagents (‘buffer’). Common mistakes that can result in invalid or misinterpreted test results include applying the incorrect sample or buffer volume, applying the buffer or blood sample to the incorrect well, or reading the RDT result outside its recommended reading period. Health workers may also misinterpret RDT results by interpreting faint test lines as negative, missing them altogether or by misidentifying the detected species in a combo test [6]. Studies show that regular health worker trainings and frequent supportive supervision can help minimize these errors [7–13].

Mobile medical applications (MMAs) can transform a mobile platform (i.e., the hardware/software environment for a mobile device such as a smart phone) into a medical device. Some MMAs can transform a smart phone into a medical device *and* take over and/or support the diagnosis function. Malaria RDT MMAs claim to accurately interpret RDT results, while microscopy MMAs claim to accurately interpret blood slides (with or without the use of additional equipment like a microscope) [14, 15]. MMAs for diagnosis are subject to regulatory control by agencies such as the United States (US) Food and Drug Administration (FDA), the European Commission (EC), the United Kingdom (UK) Medicines and Healthcare Products Regulatory Agency (MHRA) or the Australian Therapeutic Goods Administration (TGA) [16–20]. MMAs may also function as a work-flow assist to guide the end user in performing an RDT, capture and transmit images of RDTs or slides using the phone camera, perform remote evaluations of the end-user, report patient details, or assist in stock management.

The use of RDT MMAs is not yet widespread. At the time of writing this publication, their use has been limited to research and evaluation [21–25], and to date, evaluations of how these devices interpret RDT test results have been limited to the ‘Deki reader’ (MMA4 in this study) [26]. To help generate additional evidence an evaluation of five commercially available RDT MMAs was performed. The evaluation had three main objectives. First, the operational performance of RDT MMAs was assessed by comparing the diagnostic sensitivity (Sn), specificity (Sp) and agreement (Kappa) of RDT results read by MMAs to RDT results read by the human eye. Second, the repeatability of RDT MMA results was measured and third, the ability of MMAs to interpret RDT test line results was evaluated when the RDT was performed with operating errors. An overview of the product characteristics of the RDT MMAs was also provided.

## Methods

The MMA evaluation was conducted at the Malaria Branch Laboratory, the Centers for Disease Control and Prevention (CDC), in Atlanta, USA, between October 2016 and February 2017. Two full-time and one-part time staff (hereby referred to as operators) carried out the evaluation. Two operators were trained on RDT performance and result interpretation by CDC staff directly working on the RDT evaluation programme [27] prior to the start of the evaluation. MMAs came with user manuals and all three operators acquainted themselves with the use of MMAs by reviewing the instructions for use (IFU); in addition, they had online or phone trainings with the MMA designers prior to the start of the evaluation. The study protocol was reviewed and approved by the Center for Global Health (CGH) at CDC, Atlanta (CGH HSR Tracking#: 2016 -73), as non-human subjects research.

Six different RDT products from three RDT manufacturers were included that consistently met or exceeded WHO recommended procurement criteria and account for the largest share of the global RDT market [28] (Table 1). Both Pf Histidine Rich Protein 2 (HRP2) single test line and Pf/Pan HRP2/pan- *Plasmodium* lactate dehydrogenase (LDH) dual test line (‘combo’) RDTs were used. The ability of MMAs and human eye to interpret both the Pf and Pan test line results were evaluated using cultured *P. falciparum* strain 3D7 blood samples at dilutions at 20, 100, 200, 500 and a 1000 parasites (p)/µl and malaria negative blood samples. Before dilution to the target parasitaemias, the cultures were synchronized at the young trophozoïte stage with a standard protocol based on sorbitol treatment. After resuspension in a 40% haematocrit mixture of O + blood cells and AB + plasma, the parasite density was determined by two independent microscopists, based on a red cell count. Dilutions of 20, 100, 200, 500, 1000 parasites/µl were then prepared using venous donor blood uninfected by *Plasmodium* parasites (screened by microscopy and RDTs). Parasite negative whole blood samples were obtained from informed and consented volunteer donors from accredited blood banks (mostly National Blood Transfusion Centers). Blood donors were tested for malaria (microscopy, RDT) and viral infections (hepatitis B and C, HIV I and II, by ELISA).

**Table 1:** Details of RDT products included in study

Type	Product	Brand	Catalogue number*	Available lot number
Pf	CareStart™ Malaria HRP2 (Pf)	Access Bio Inc.	RMOM-02571, RMOM‐05071	MO16G63
Pf/Pan	CareStart™ Malaria HRP2/pLDH (Pf/PAN) COMBO	Access Bio Inc.	RMRM-02571, RMRM‐05071	MR16G61
Pf	SD BIOLINE Malaria Ag P.f (HRP2)	Abbott	05FK50	05CDB086A
Pf/Pan	SD Bioline Malaria Ag Pf/Pan	Abbott	05FK60	05EDB020A
Pf	First Response® Malaria Ag *P. falciparum* (HRP2) Card Test	Premier Medical Corporation Private Limited	I13FRC25, 13FRC30	56F1116S
Pan	CareStart™ Malaria pLDH (PAN)	Access Bio Inc.	RMNM-02571, RMNM-05071	MN16G62

Although most clinical malaria infections manifest themselves with much higher parasite loads, a sample of 20 p/µl to induce low band intensity reactivity was included to see how the human eye and the readers would compare when reading such low band intensities. Over sixteen hundred RDTs (1625) of each product were shipped from the manufacturers’ site to the US Centers for Disease Control via airfreight at ambient temperature and without temperature monitoring. Four RDTs of each RDT product were then shipped to the MMA manufacturers for calibration purposes via ground/air freight under the conditions described previously. No specific instructions were provided to the manufacturers on what or how to perform the calibration.

### Inclusion and exclusion criteria for MMAs

MMAs were identified and selected based on snowball sampling. RDT MMAs were included if they met the following specifications: (a) produce qualitative test results (positive, negative or invalid test result); (b) are small and light enough to be portable/handheld; (c) have a battery power option available; (d) come in dust-free packing; (e) require limited or no additional equipment; (f) require minimal training so that lay operators can be trained within 2 hours and (g) cost less than USD $3,000. Potential RDT MMA technologies were excluded when MMAs (a) required a cold chain and (b) were not available within the timeline set to perform the study. MMAs were shipped to CDC in Atlanta via routine mail delivery using FedEx. Table 2 provides a visual and a short description of the MMAs included in the study.

**Table 2:** List of MMAs included in evaluation

No.	Short form	MMA System type	Product Name	Manufacturer	Country	Training method	Visual
1	MMA1	Open system ^a^	GSID Reader (Beta)	Global Solutions for Infectious Diseases (GSID)	USA	Documentation review	
2	MMA2	Open system	IDA Malaria mobile reader	ISTOC Oy	Finland	Documentation review	
3	MMA3	Closed system	opTrilyzer	opTricon Gmbh	Germany	Telephonic/online	
4	MMA4	Closed system	Deki Reader	Fio Corporation	Canada	Documentation review	
5	MMA5	Closed system	HRDR-200 Rental	Cellmic LLC	USA	Documentation review	

^a^MMA1 and MMA2 are open system MMAs as they do not control for the amount of external light falling on the RDT or fix the distance between the MMA camera and the RDT. MMA3, MMA4 and MMA5 are closed system MMAs.

### Evaluation procedures

**Fig. 1:**
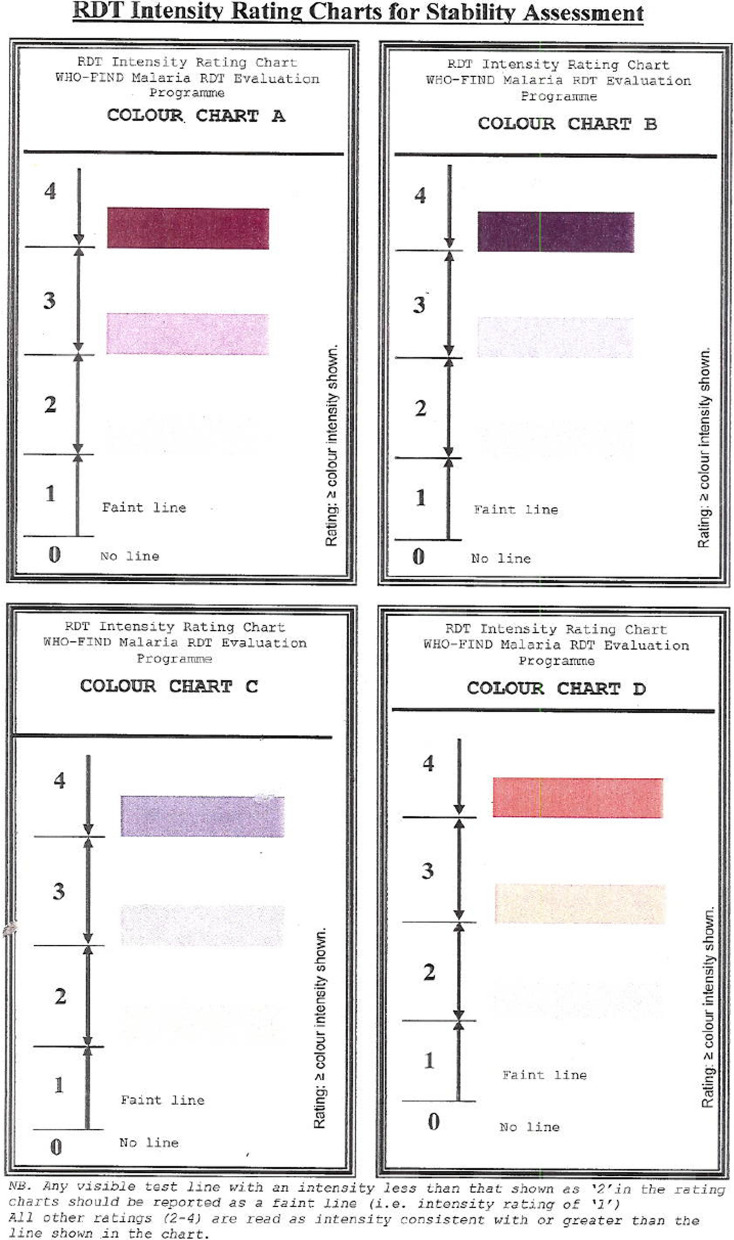
RDT Intensity Rating Charts for Stability Assessment*. *Shades are different from the actual template and is influenced by each or all of quality of printer, scanner and computer monitor resolution. Operators used a copy that was available in the CDC Malaria lab

To achieve the first objective, operator 1 performed the RDT according to manufacturers’ instructions, read and recorded the result as positive, negative, invalid, and scored the RDT control and result line(s) intensity. Intensities were scored using a scale from 0 to 4 using the band intensity template from the WHO malaria RDT Product Testing as a guide [29] (Fig. 1); 0 corresponded to the absence of a line. Operator 2 used the MMA to read the same RDT, immediately or within 1–2 mins, and recorded results. Both the human eye and MMA result interpretation of the RDT were performed within the specified reading time of each RDT product. Operator 1 and 2 were both blinded to the sample type (*P. falciparum* or negative) and parasite density. Operator 2 was blinded to the RDT result noted by operator 1 and vice versa. Samples used on any particular day were not re-used.

For the second objective, repeatability was calculated. Ten RDTs of each of the six RDT types were performed using negative samples and samples with each of the five parasite densities. The MMAs then read each of the RDT type/sample combinations multiple (ten) times. Repeatability for each of the RDT type/sample combinations was defined as the number of true positive or negative results following the first true positive or true negative result out of the total of ten read outs performed.

For the third objective, seven different types of RDT protocol errors were intentionally made while performing the RDT. Errors were induced by adding (1) excess, (2) insufficient, or (3) no blood to the sample well; adding (4) excess, (5) insufficient, or (6) no buffer to the sample well; and finally (7) dropping buffer or blood in the incorrect sample well. Samples containing 1000 parasites/µL were used for this purpose. The seven different errors were performed once on each of the six different RDT products. MMAs were assessed, for correctly categorizing and identifying the results as errors, compared to the human eye. Human operators were trained on identification of errors and had a pictorial reference of different errors at their disposal.

Finally, an overview of the product characteristics of MMAs was provided by noting down observations about the use of the MMAs during and after the course of the evaluation and by reviewing MMA user manuals shared by the designers.

### Statistical analysis

The required sample size was calculated using a non-inferiority study protocol with 80% power, 5% significance and the joint hypotheses that the RDT Sn when read by the MMA is not decreased by more than 90% compared to when read by the human eye, and the false positive fraction (1-specificity) is not more than twice that of the human eye. It was assumed that the performance of the RDTs varies by parasite concentration in the sample. It was assumed that samples at 1000, 500, and 200 parasites/µl would have 95% Sn when read by the human eye (i.e., the human eye), while samples at 100 and 20 parasites/µl would have a RDT Sn of 90% when read by the human eye. RDT Sp when read by the human eye was assumed to be 95%. Based on these assumptions, it was estimated that a total of 1610 samples were required for each RDT, with a target profile of 49.7% negative samples, and 18.6%, 13.0%, 6.2%, 6.2%, and 6.2% of samples at 20, 100, 200, 500, and 1000 parasites/µl, respectively. Data was recorded on paper data collection sheets and later entered in MS Excel spreadsheets (Microsoft Corporation, Seattle, Washington, USA). Double data entry was performed. Data analysis was performed using R (version 3.4.1).

The primary analysis was conducted using sample reactivity (positivity) as the reference method to determine diagnostic performance characteristics (Sn, Sp, and agreement) of either the Pf or Pan test line interpretation, by the human eye and the MMA. Diagnostic performance characteristics (Sn, Sp) of both Pf and Pan test lines were also analysed for the two ‘combo’ RDTs included in the evaluation (the interpretation of a positive *P. falciparum* result for a Pf/Pan combo test includes either *or* both the Pf and Pan test line to be positive). A Bayesian framework was used to estimate Sn/Sp and agreement (Cohen’s kappa), and to provide 95% credible intervals (CI). The probability of the sample to be truly positive was derived and used to compute sensitivity and specificity with a mixed model that accounts for fixed effects, i.e. concentration & readers (including human eye) and random effects, i.e. RDT types, using a logistic regression.

## Results

Overall, 9332 RDTs were performed across six RDT products using five different sample concentrations and negative samples. The number of specimens tested against each RDT ranged from 1510 to 1570, with the target sample profile maintained within ± 0.6%.

### Sensitivity and specificity

**Fig. 2:**
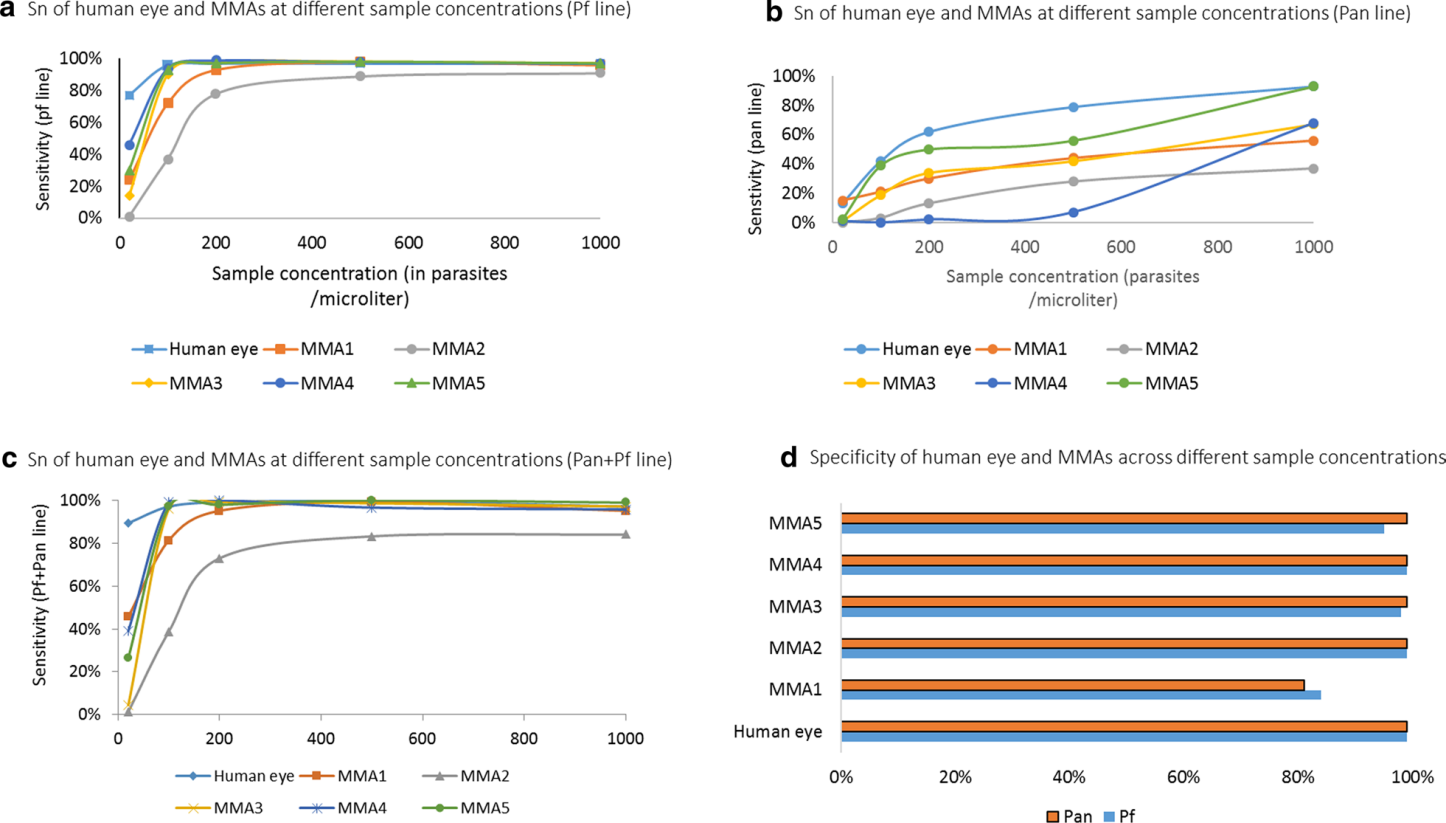
**a** Sn of human eye and MMAs at different sample concentrations (Pf line). **b **Sn of human eye and MMAs at different sample concentrations (Pan line).  **c** Sn of human eye and MMAs at different sample concentrations (Pan+Pf line). **d** Specificity of human eye and MMAs across different sample concentrationsSpecificity of human eye and MMAs across different sample concentrations

The RDT Sn of the *P. falciparum* (HRP2) line across all Pf only and Pf/Pan RDTs, when read by the human eye ranged from 77% (95% CI, 57–86%) at 20 p/µl to 99% (95% CI 99–100%) at 1000 p/µl. Malaria RDT Sn when read by the best performing MMA (MMA4) ranged from 47% (95% CI 31–64%) at 20 p/µl to 99% (95% CI 98–99%) at 1000 p/µl. Malaria RDT Sn when read by other MMAs ranged from 4% (95% CI, 2–8%) at a density of 20 p/µl to 98% (95% CI 97–99%) at a density of 1000 p/µl. Malaria RDT Sp when read by the human eye was 99% (95% CI 98–100%). Malaria RDT Sp read by the MMAs ranged from 86% (95% CI 70–94%) for MMA1 to 99% (95% CI 97–100%) for MMA2 and MMA4 (Fig. 2d). Malaria RDT Sn read by MMAs rapidly increased across MMAs with density levels at 200 p/µl and above (Fig. 2a ).

Sensitivity and Sp scores for the Pan line were lower for RDTs read by the human eye or the MMAs (Fig. 2b). The RDT Sn read by the human eye ranged from 27% (95% CI 6–67%) at 20 p/µl to 95% (95% CI 69–98%) at 1000 p/µl (Fig. 1b). The RDT Sn when read by the best performing MMA (MMA5) ranged from 10% (95% CI 2–54%) at a density of 20 p/µl to 85% (95% CI 44–94%) at a density of 1000 p/µl. RDT Sn when read by other MMAs ranged from 9% (95% CI 2–53%) at a density of 20 p/µl to 84% (95% CI 43–94%) at a density of 1000 p/µl. The RDT Sn and Sp for positive *P. falciparum* result were also analyzed interpreting either *or* both the Pf and Pan test line being positive in the two Pf/Pan RDTs included in the study. Malaria RDT Sn when read by the human eye ranged from 89% (95% CI 70–97%) at 20 p/µl to 99% (95% CI 100–100%) at 500 p/µl. The Sn when read by the best performing MMAs (MMA4, MMA5) ranged from 26% (95% CI 19–71%) at a density of 20 p/µl to 100% (95% CI 93–100%) at a density of 500 p/µl. Malaria RDT Sn when read by other MMAs ranged from 1% (95% CI 1–10%) at a density of 20 p/µl to 99% (95% CI 94–100%) at a density of 500 p/µl. (Fig. 2c). Additional analysis showed that Sn or Sp results did not significantly differ by RDT product brand.

### Agreement

For the Pf line (in Pf only and Pf/Pan RDTs), the agreement (median kappa) of the MMAs compared to the human eye ranged from 0.46 (95% CI 0.44–0.47) for MMA2 to 0.85 (95% CI 0.84–0.86) for MMA4 across all sample densities. In other words, MMA2 showed moderate agreement and MMA4 nearly complete agreement with the human eye [30]. For the Pan line (in Pan only and Pf/Pan RDTs), the median kappa of the MMAs ranged from 0.18 (95% CI 0.15–0.21) for MMA1 to 0.66 (95% CI, 0.63–0.68) for MMA5. Tables 3 and 4 provide an overview of the agreement across all densities for the Pf and Pan line, respectively.

**Table 3:** Modelled median Kappa (reference: human eye) for Pf line

Readers	Observed	Modelled		
		Median	95% LCI	95% UCI
MMA1	0.514	0.514	0.498	0.529
MMA2	0.459	0.459	0.444	0.473
MMA3	0.689	0.688	0.675	0.702
MMA4	0.850	0.850	0.835	0.862
MMA5	0.737	0.737	0.716	0.758

**Table 4:** Modelled median Kappa (reference: human eye) for Pan line

Readers	Observed	Modelled		
		Median	95% LCI	95% UCI
MMA1	0.179	0.180	0.153	0.206
MMA2	0.296	0.296	0.271	0.323
MMA3	0.576	0.576	0.552	0.601
MMA4	0.483	0.483	0.416	0.549
MMA5	0.656	0.656	0.631	0.681

**Fig. 3:**
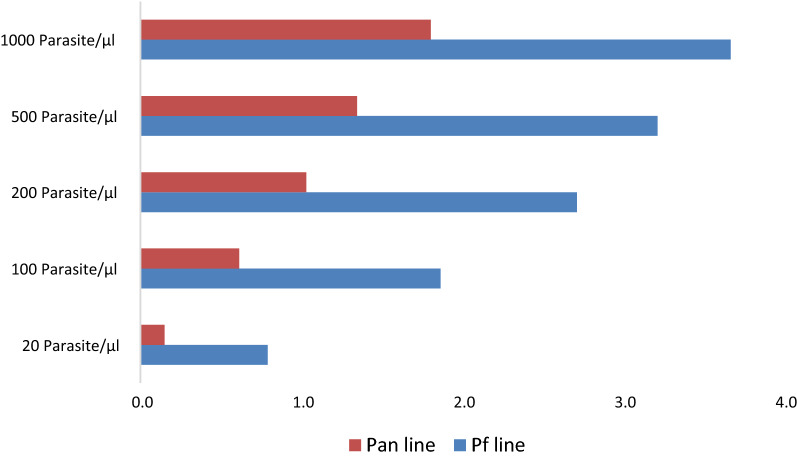
Average test line intensities or each by sample concentrations

The test line intensity (as rated by the human eye using the band intensity template)) was found to have increased with the sample concentration. Overall, the average Pf line intensity across RDT types was higher than the average Pan line intensity for identical sample concentrations: For Pf and Pan lines, with negative samples, the average line intensity was 0. For the Pf line, the average line intensity was 1 with samples at 20 p/µl, increasing to an average intensity of 4 at 1000 p/µl. For the pan line, lower average line intensities were observed; with a maximum average line intensity of 2 observed at 1000 p/µl. Figure 3 provides an overview of the average test line intensities or each of the sample concentrations.

### Repeatability

Repeatability of MMAs reading the Pf line varied between 0%–100% depending on RDT type/sample combinations. In negative samples, repeatability was 100% or close to 100% for all MMAs evaluated (i.e., MMAs reported ten consecutive negative results). Repeatability at 20 p/µl was 0% for all MMA types across all RDT types (i.e., none of the MMAs reported a positive result), except for MM3 (100% for the First Response Pf RDT) and MMA1 (100% for the Carestart Pf/Pan RDT and SD Bioline Pf/Pan RDT; 60% for the Carestart Pf RDT). Repeatability of all MMAs increased at higher sample densities irrespective of RDT type: at a density of 200 p/µl and above repeatability was 90% or 100% for all MMAs evaluated.

**Fig. 4:**
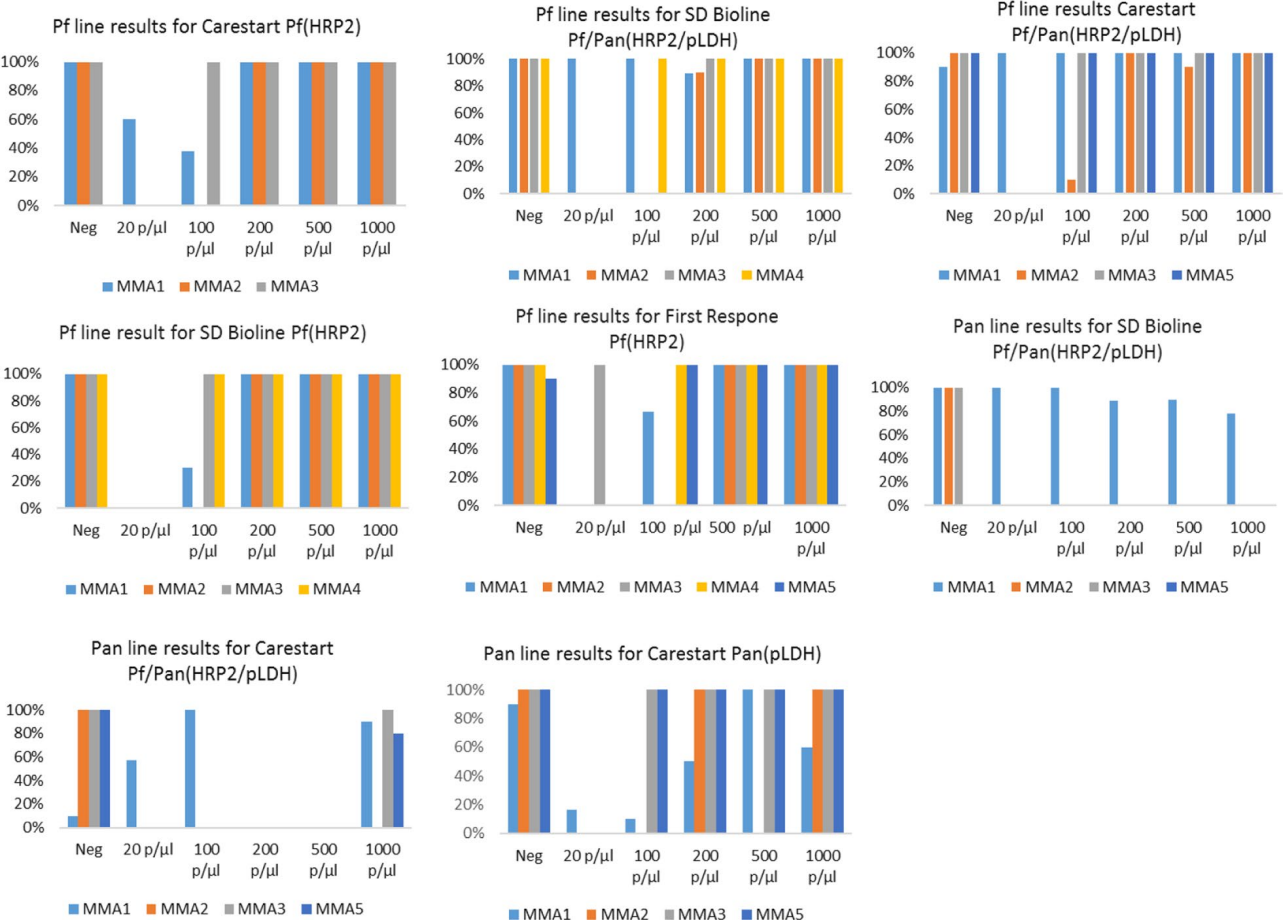
Repeatability scores by RDT type and sample density

Repeatability of MMAs reading the pan line of the two combo RDTs (SD Bioline Pf/Pan RDT, Carestart Pf/Pan RDT), was 0% across all sample densities, except for MMA1. For MMA1, repeatability ranged from 0% in a density of 200 p/µl for the Carestart Pf/Pan RDT to 100% across densities for the SD Bioline Pf/Pan RDT. Repeatability of MMA2, MMA3 and MMA5 for the Pan only RDT (Carestart Pan RDT) ranged from 0% for samples at 20 p/µl to 100% at the higher dilutions. Repeatability scores for MMA1 ranged from 10% at 100 p/µl to 100% at 500 p/µl (Fig. 4).

### Introduced errors in RDTs

**Fig. 5:**
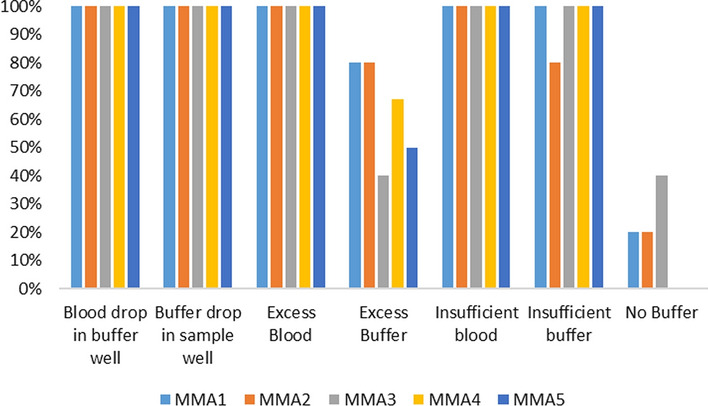
Agreement between the human eye and the MMAs for each of the induced errors

The ability of MMAs to detect and categorize RDTs that were performed with errors was also measured. All MMAs had a classification system for errors that classified results as either *invalid* or *no control line detected* when anomalies were observed. In adding too much (error #1) or too little blood (error #2) of a sample of 1000 p/µl to the RDTs, neither the MMAs nor the human eye detected any errors, but generally interpreted the line as positive. Blood of a positive *P. falciparum* sample (1000 p/µl) was added in the buffer well instead of the blood well and vice versa (error #3 and #4). In both instances, again, neither the MMAs nor the human eye were able to detect the errors and interpret the line as positive. Similar results were obtained when too little buffer was applied (error #5). However, when excess buffer (error #6) or no buffer was used (error #7), all MMAs interpreted the result as negative, rather than showing an error classification (i.e., invalid or no control line) like the human eye did. Figure 5 provides an overview of the agreement between the human eye and the MMAs for each of the induced errors.

Table 5 provides an overview of the product characteristics as observed during the evaluation. All MMAs came with IFU but none of the IFUs included a troubleshooting guide. A thirty to sixty-minute review of the IFU was sufficient to operate the MMAs. MMAs required two to nine steps to generate a result, taking an average time of 16 seconds (ranging from seven seconds (MMA2) to 23 seconds for MMA4) as compared to an average of two seconds for the trained human eye. MMAs needed a battery recharge once or at most twice a day with continuous use over a period of 8 h. MMAs seemed durable during the evaluation, although some MMAs began to show minor signs of wear and tear after extended periods of constant use (i.e., a door magnet for the RDT tray of MMA5 came undone).

**Table 5:** Operational Characteristics of MMAs

Operationac characteristics	MMA1	MMA2	MMA3	MMA4	MMA5
1. Quality of the Instructions for Use (IFU) for MMA					
a. Instructions (0—not present, 1—present)	Present	Present	Present	Present	Present
b. Pictures/diagrams of method (0—no pictures or 1—pictures present)	Picture present	Picture present	Picture present	Picture present	Picture present
c. Pictures diagrams of result (0—no pictures or 1—pictures present)	Picture present	Picture present	Picture present	Picture present	Picture present
d. Trouble shooting guide (0—not present, 1—present)	Not present	Not present	Not present	Not present	Not present
2. Timing					
a. Steps required	7	2	9	4	5
b. Total time to result (in seconds)	16 to 17.5	7	17 to 18	23 to 24	17 to 18
3. Language of instructions (0—Non- English only; 1—English 2- English and other)	1	2	2	2	1
4. Calibration					
a. Required (Yes/No)	No	No	No	Yes	No
b. No. of steps	No	No	No	4	No
c. Number of Controls	No	No	No	1	No
5. Training					
a. Recommended time for training: More than 1 day— 0, Less than 6 h—1, 2 hours or less—2	Less than 6 hours	Less than 6 hours	Less than 6 hours	Less than 6 hours	Less than 6 hours
b. Mode of training: In-person training—0, Phone based only—1, Skype/Video chat based —2, Interactive web-based — 2	Skype/video chat based or interactive web-based	Skype/video chat based or interactive web-based	Skype/video chat based or interactive web-based	Skype/video chat based or interactive web-based	Skype/video chat based or interactive web-based
6. Other					
a. Portability/Mobility (0—not portable, 1—portable)	Portable	Portable	Portable	Portable	Portable
b. Frequency of Indeterminate result/error (0–4–5 errors, 1–2–3 errors, 2–0–1 errors)	0	0	2	1	1
c. Maintenance Requirements (0—high maintenance requirements, 1—no maintenance needed)	No maintenance needed	No maintenance needed	No maintenance needed	No maintenance needed	High maintenance required

## Discussion

To evaluate the performance of commercially available MMAs, an evaluation was conducted by comparing RDT results read by MMAs to RDT results read by the human eye. At parasite densities of 100 p/µl and higher, the RDT Sn when read by both the human eye and MMAs was high (close to 100%) for the Pf line, with the exception of MMA1 (Sn of 72%) and MMA2 (Sn of 37%). These two MMAs were the only ‘open’ MMAs included in the evaluation where the amount of light falling on the RDT is not controlled. At a lower parasite density (20 p/µl), the RDT Sn was low but the value was higher when read by the human eye compared to when read by the MMAs. Specificity was close to 100% for RDTs, irrespective of whether it was read by the human eye or MMA, except for MMA1 (82%). In identifying the Pan line, the RDT Sn using both the human eye and the MMAs was generally poor, with lower Sn when read by the MMA.

The MMAs scored poorly on repeatability at low densities for the Pf line and across all densities on the pan line, but 100% at high densities for the Pf line (i.e., 500 or 1000 p/µl) and with negative samples. Kappa agreement between human eye and MMAs varied across all samples ranging from 0.459 for MMA2 (low) to 0.850 (high) for MMA4. Further analysis showed that agreement and repeatability results were lower across all MMAs for low density examples (20 p/µl).

The ability of MMAs to read and identify RDTs conducted with operating errors was also assessed resulting in a suboptimal presentation of the test strip. Anomalies like incomplete clearing or red background frequently occur. The Round 8 WHO malaria RDT product evaluation found at least one anomaly in over half (19/35) of all RDT products evaluated [29]. The evaluated MMAs only had a rudimentary error classification system in place (invalid, no control line detected) and in most cases did not pick up on any of the errors induced (nor did the human eye).

Operators also provided an overview of the product characteristics of the MMAs. The operators found the MMAs easy to use and experienced few problems. During the evaluation, there were some instances of hardware and software challenges, which were resolved in a couple of days, due to ease of communication and relative proximity to the service centers of the designers, as the study took place in a reference lab and not in a field setting. However, resolving technical issues may be more difficult in field settings where international communication is limited or there are differences in time zones.

The results from this study demonstrate that the ability of MMAs to correctly identify positive samples is closely correlated with observed line intensity: the lower the test line intensity (as scored by the human eye), the lower the level of accuracy of the MMAs or agreement with the human eye. The relatively poor performance of the MMAs in recognizing low intensity lines could be secondary to a number of factors. A lack of calibration of MMAs at various line intensities or setting the intensity cut off point for positive tests too high (by design) could have caused the MMAs to report false negatives. Other reasons that may have affected the result interpretation include the amount of light falling on the RDT cassette (especially relevant in an open system like MMA1 or MMA 2, as they do not control for the amount of light entering the system); or RDT surface properties (matte vs. shiny finish) that may affect the quality of images captured.

Results from our evaluation are similar to those found in other recent evaluations of the Deki reader, MMA4 in this study [22–25, 31, 32]. Rather than using cultured samples in a laboratory setting, these field evaluations compared RDT test results of patients suspected of having P. *falciparum* malaria read by the human eye and the Deki reader to that of a reference method (i.e., microscopy). In Tanzania, the human eye (trained lay workers) and the Deki reader achieved a similar Sn of around 94% in interpreting 1293 RDT results compared to microscopy. At parasite levels below 200 p/µl, both the human eye and the Deki reader achieved a lower but similar Sn of 69% [23]. In Uganda, researchers found high agreement between human eye and the Deki reader (98.9%) in comparing 566 RDT results [31]. In a study in Kenya, additional functionality programmed on the Deki reader allowed for real-time feedback to community health workers (CHWs) performing the RDT [32]. When the Deki reader determined results were invalid, it also provided information about the source of the error, including errors such as too much blood, too little buffer or placing the sample in the wrong well. Across all studies, researchers reported that health workers welcomed the Deki reader and found the device relatively easy to operate, although network connectivity or low battery power limited the ability to adequately operate the reader at times. The version of the Deki reader used in this study did not have the error classification functionality.

This study suggests that the potential benefit of using MMAs in malaria case management may not be in improving overall accuracy in diagnosing malaria but rather in collecting images and other test information that may help in identifying and classifying RDT anomalies or errors in specific cases that may otherwise go unnoticed in resource constrained settings where post-market surveillance for RDTs as well as general quality assurance occurs infrequently or not at all. MMAs could capture, monitor, and report this information in real time, allowing for immediate follow up if required, serving as a quality assurance tool. However, this benefit should be weight against the lower sensitivity of RDTs when read by MMAs at lower parasite densities, or on pan-only RDTs.

## Limitations

The evaluation had a number of limitations. First, only cultured *P. falciparum* samples were used, rather than clinical *P*. *falciparum* and *P. vivax* samples to evaluate the Pf and Pan lines. However, because RDTs are designed to detect *P. falciparum* with either HRP2 or pLDH the results are not believed to be influenced to a great extent by the choice of culture-derived samples. Second, the evaluation was limited to the result interpretation capability and did not evaluate any other functionalities that MMAs may have had (or could be programmed to have). The study only used 93.4–97.5% of the target number of samples per RDT, although the profile of the samples was maintained. The higher than assumed Sp for the human eye reduces the power to detect significant differences in Sp. The higher than assumed Sn of the human eye for samples with ≥ 100 p/µl compensates for the lower number of samples to maintain statistical power at higher concentrations, while the lower than assumed Sn for 20 p/µl and actual samples size means decreased Sn among the MMAs of more than 85% compared to the human eye should be detected with 80% power at 5% significance.

## Conclusions

The results of the study show that at *P. falciparum* densities typically expected with clinical malaria (i.e. above 100p/µl), some MMAs can perform as well as the trained human eye in detecting the Pf line of an RDT. At low band intensities, and with non- falciparum malaria, the trained human eye outperforms the results interpretation ability of MMAs. Future development and research efforts should focus on improving the band/line detection for low band intensities and conducting field evaluations that include other MMA functionalities, including error classifications, to inform whether or not MMAs can serve as a quality assurance tool in malaria case management.

## Data Availability

The datasets used and/or analysed during the current study are available from the corresponding author on reasonable request.
